# A conserved role for Notch signaling in priming the cellular response to Shh through ciliary localisation of the key Shh transducer Smo

**DOI:** 10.1242/dev.125237

**Published:** 2015-07-01

**Authors:** Magdalena Stasiulewicz, Shona D. Gray, Ioanna Mastromina, Joana C. Silva, Mia Björklund, Philip A. Seymour, David Booth, Calum Thompson, Richard J. Green, Emma A. Hall, Palle Serup, J. Kim Dale

**Affiliations:** 1 Division of Cell and Developmental Biology, College of Life Sciences, University of Dundee, Dow Street, Dundee DD1 5EH, Scotland, UK; 2 The Danish Stem Cell Center, Faculty of Health Sciences, University of Copenhagen, Blegdamsvej 3B, Copenhagen DK-2200, Denmark; 3 MRC Human Genetics, Institute for Genetics and Molecular Medicine, University of Edinburgh, Edinburgh EH4 2XU, UK

**Keywords:** Notch, Shh, Embryo, Chick, Mouse, Cilia, Notochord, Floor plate, P3 progenitors

## Abstract

Notochord-derived Sonic Hedgehog (Shh) is essential for dorsoventral patterning of the overlying neural tube. Increasing concentration and duration of Shh signal induces progenitors to acquire progressively more ventral fates. We show that Notch signalling augments the response of neuroepithelial cells to Shh, leading to the induction of higher expression levels of the Shh target gene *Ptch1* and subsequently induction of more ventral cell fates. Furthermore, we demonstrate that activated Notch1 leads to pronounced accumulation of Smoothened (Smo) within primary cilia and elevated levels of full-length Gli3. Finally, we show that Notch activity promotes longer primary cilia both *in vitro* and *in vivo*. Strikingly, these Notch-regulated effects are Shh independent. These data identify Notch signalling as a novel modulator of Shh signalling that acts mechanistically via regulation of ciliary localisation of key components of its transduction machinery.

## Abstract (precis)

**Highlighted article:** Shh signalling controls dorso-ventral cell fate in the neural tube. Notch regulates ciliary architecture and localisation of key Shh pathway components, thus sensitising cells to Shh.

## INTRODUCTION

The notochord is the source of a signal, Sonic Hedgehog (Shh), that patterns the dorsoventral aspect of the neural tube (Echelard et al., 1993; Krauss et al., 1993; Marti et al., 1995). The response of neural progenitors is dependent on both the concentration and duration of Shh signalling to which they are exposed (reviewed by Cohen et al., 2013; Briscoe and Novitch, 2008; Briscoe and Thérond, 2013). This leads to the induction and spatial distribution of distinct transcription factors in different progenitor pools along the dorsal-ventral axis of the neural tube. The most ventral populations are floor plate and p3 progenitors (which will give rise to V3 interneurons). In the absence of Shh signalling, these cell types do not develop. Floor plate induction initially requires exposure to a high burst of Shh but full floor plate maturation requires that these cells then attenuate their response to Shh (Ribes et al., 2010). By contrast, maintenance of Shh signalling is required for the full differentiation of the p3 progenitors.

It is well established that Shh signal transduction requires primary cilia (Huangfu et al., 2003; reviewed by Sasai and Briscoe, 2012). In the absence of Shh ligand, the transmembrane receptor Patched1 (Ptch1) is located in the base of the cilia and represses the pathway by binding and inhibiting the ciliary localisation of Smoothened (Smo) (Taipale et al., 2002; Rohatgi et al., 2009, 2007; Sasai and Briscoe, 2012; Chen et al., 2011; Milenkovic et al., 2009; Stamataki et al., 2005; Briscoe and Thérond, 2013). Activation of the pathway is achieved by binding of Shh to Ptch1, which releases the inhibition on Smo, allowing it to translocate into the cilia. Smo prevents proteolytic cleavage of the Gli transcription factors, so that full-length Gli translocates to the nucleus to activate target gene transcription. It is possible that other signalling pathways may interact with the Shh pathway by regulating ciliary translocation of these key transduction components.

The Notch pathway plays a key role in various aspects of patterning and cell fate choice during neurogenesis (Henrique et al., 1995; Louvi and Artavanis-Tsakonas, 2006; Pierfelice et al., 2011; Hori et al., 2013; Guruharsha et al., 2012), such as balancing numbers of progenitor cells with that of differentiating neurons through lateral inhibition/specification (Henrique et al., 1995; Pierfelice et al., 2011) and regulating binary cell fate choice of progenitors as they differentiate into different neuronal subtypes (Okigawa et al., 2014). Both receptor and ligands are membrane-bound proteins so the pathway is activated by cell-cell communication. Upon activation by the Delta/Serrate ligands in adjacent cells, the Notch receptor undergoes a number of proteolytic cleavage events, the last of which is mediated by a γ-secretase enzyme complex that cleaves the intracellular domain of Notch (NICD). NICD translocates to the nucleus where it binds to the obligate transcription factor of the pathway, RBPJ, and creates a dual binding interface for the mastermind-like (Maml) family of proteins that are essential components of the transcriptional activation complex. This ternary complex is essential for transcriptional activation of Notch target genes.

Recent reports have demonstrated enrichment of Notch signalling components in primary cilia and that aberrations in ciliogenesis impact on activation of the Notch pathway (Leitch et al., 2014; Ezratty et al., 2011). Despite extensive studies on the role of Shh and Notch pathways in patterning and development of the central nervous system, and the intriguing fact that primary cilia mediate efficient signalling for both pathways (at least in some contexts), nothing is known about the potential crosstalk between these pathways during establishment of the dorsoventral pattern of progenitor domains across the neural tube.

Here, we report a novel role for Notch in augmenting the response of neural progenitors to Shh in the chick and mouse neural tube, and provide insight into the establishment of floor plate and P3 identity. Using gain- and loss-of-function assays, we show that Notch is required for cells to acquire the most ventral cell fate in response to Shh but that attenuation of Notch signalling is equally important for these cells to fully differentiate as floor plate. Strikingly, we show that Notch activation promotes Shh-independent accumulation of Smo within cilia, leads to elevated levels of full-length Gli3 and formation of longer cilia. Together, the data suggest Notch acts mechanistically to prime neural progenitor cells to respond to Shh through changing ciliary architecture and localisation of Smo to the cilia.

## RESULTS

### Notch activation mirrors Shh target gene expression in floor plate and P3 domains

**Fig. 1.**
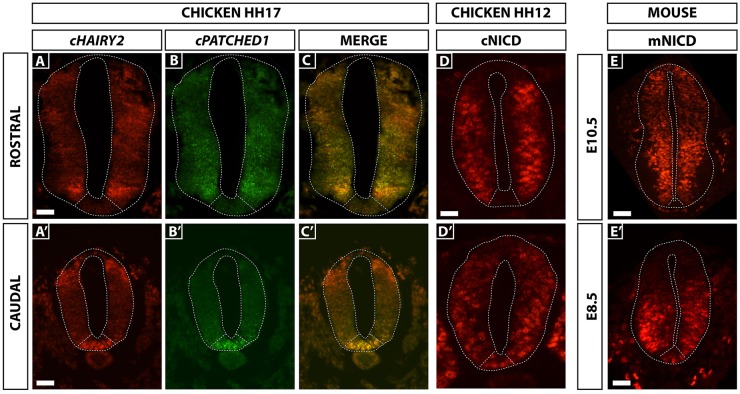
**Notch activation mirrors Shh target gene expression in floor plate and P3 domains.** (A-C′) Sections showing *cHairy2* (A,A′) and *Ptch1* (B,B′) expression in the same neural tube, analysed by fluorescent *in situ* hybridisation. Scale bars: 30 µm. (D-E′) Transverse sections of chick (D,D′) and mouse (E,E′) embryos showing the profile of NICD by immunohistochemistry. Scale bars: 20 µm in D; 50 µm in E; 30 µm in E′). (A′-D′) Sections through caudal, lumbar regions of the neuraxis. (A-E) Sections through more developmentally mature, brachial regions of the neuraxis. (C,C′) Merged images of *cHairy2* and *Ptch1* mRNA expression. *cHairy2* is also expressed in more the dorsal neural tube (Broom et al., 2012).

We previously showed that the Notch target gene *cHairy2* is expressed in Hensen's node and axial tissues as they leave the node in HH stage 5-11 chick embryos, where it is co-expressed with the earliest floor plate marker *cFoxa2* (Gray and Dale, 2010). As development proceeds *cHairy2* expression in the ventral neural tube mirrors that of the Shh target *Ptch1*, both spatially and temporally: expression is high in the floor plate and P3 progenitor domains in the caudal neuraxis, whereas, in more anterior developmentally mature regions, expression is downregulated in the floor plate but maintained in the adjacent P3 progenitor domain (Fig. 1A-C′; Ribes et al., 2010). Immunohistochemistry for the cleaved activated form of the Notch1 receptor, NICD, in mouse and chick, reveals that the profile of NICD production coincides with *cHairy2* expression in the floor plate and P3 domain, in addition to the previously reported NICD activity in progenitors lining the lumen of the neural tube (Fig. 1D-E′). The Notch target *Hes1*, orthologue of *cHairy2*, is also expressed in the mouse ventral neural tube (data not shown; Sasai et al., 1992; Jouve et al., 2000). Thus, NICD production and *cHairy2/Hes1* expression occur at the right time and place to play a role in floor plate development.

### Shh induces *cHairy2* expression in I-LNP in a Notch-dependent manner

**Fig. 2.**
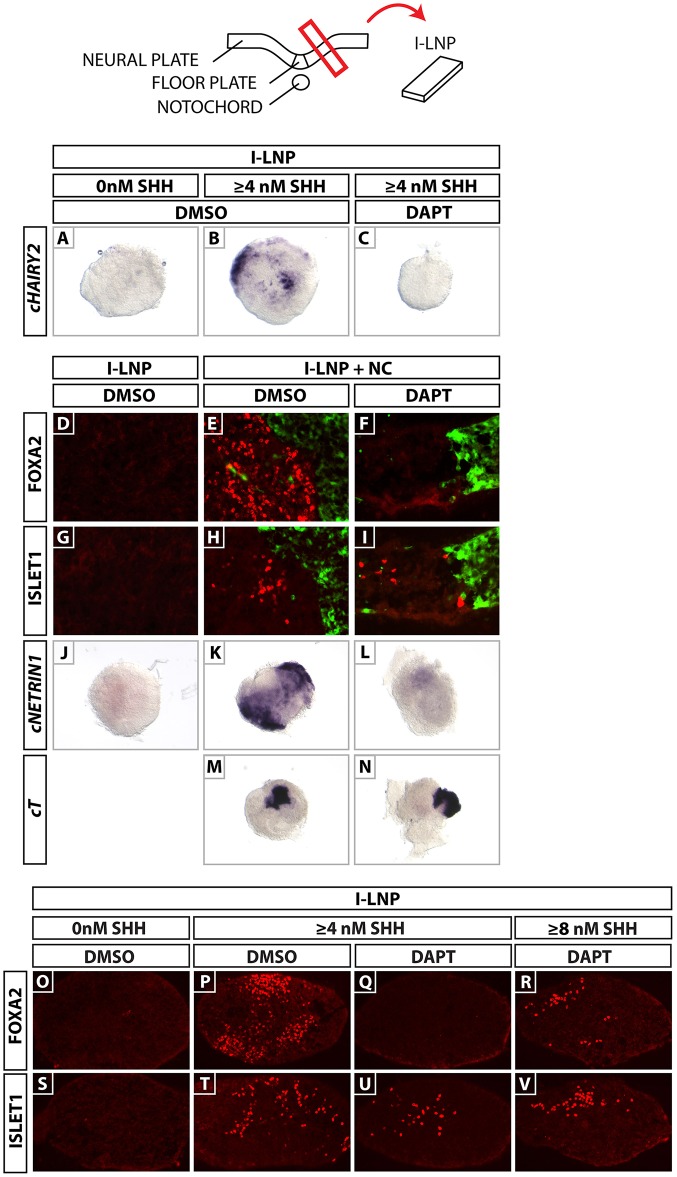
**Notch inhibition prevents floor plate but not motor neuron induction by notochord/ShhN.** Schematic of the I-LNP dissection assay. (A) I-LNPs do not express *cHairy2*. (B) ShhN induces *cHairy2* in I-LNP. This is inhibited by DAPT (C). (D-N) I-LNPs cultured alone (D,G,J) or in contact with a GFP^+^-notochord in DMSO (E,H,K,M) or DAPT (F,I,L,N). Serial sections analysed for Foxa2 (E,H) or Isl1 (F,I). (D,G,I,M) Isolated I-LNP does not express Foxa2 or Isl1. Notochord induction of Foxa2 (E) is inhibited by DAPT (F). Isl1 induction is not affected (H,I). (J) Isolated I-LNP does not express *cNetrin1*. Notochord induction of *cNetrin1* (K) is inhibited by DAPT (L). (M,N) *cT* expression is unaffected by DAPT. (O-V) Sections of I-LNP explants. I-LNP explants cultured in 4 nM ShhN expressed both Foxa2 (P) and Isl1 (T). DAPT exposure prevented Foxa2 expression (Q) but maintained Isl1 (U). I-LNP explants cultured in 8 nM ShhN plus DAPT expressed both Foxa2 (R) and Isl1 (V). I-LNP, intermediate lateral neural plate tissue; *cT*, *cBrachyury*.

To examine whether *cHairy2* transcription is Shh dependent we microdissected intermediate lateral neural plate (I-LNP) explants, which would never normally express *cHairy2*, from HH stage 6 chick embryos and cultured them in the presence/absence of recombinant Shh protein (ShhN). I-LNP alone did not express *cHairy2* (*n*=0/3; 2 LNPs per explant; Fig. 2A). However, exposure to 4 nM ShhN (the concentration required to induce floor plate markers; Ericson et al., 1996) induced *cHairy2* (*n*=12/13; 2 LNPs per explant; Fig. 2B). Using the γ-secretase inhibitor DAPT (Dale et al., 2003; Morohashi et al., 2006) to inhibit Notch signalling, we found that 50 µM DAPT inhibited Shh induction of *cHairy2* (*n*=9/9; 2 LNPs per explant; Fig. 2C). Thus, Shh induces *cHairy2* in the neuroepithelium in a Notch-dependent manner. This suggests that Shh-dependent onset of *cHairy2* expression is part of the response of these midline cells to becoming floor plate.

### Notch inhibition prevents notochord induction of Foxa2

To address whether Notch plays a role in Shh-mediated floor plate induction by notochord, we micro-dissected HH stage 6 chick I-LNPs and cultured them with a HH stage 6 notochord from GFP-expressing chick embryos. Explants co-cultured in DAPT showed no *cNetrin1* or Foxa2 expression (*n*=12/14 and *n*=5/5, respectively; Fig. 2F,L) compared with controls (*n*=5/5; Fig. 2E; *n*=16/16; Fig. 2K). Surprisingly, DAPT did not affect induction of the motor neuron marker Islet1 (Isl1) (controls *n*=5/5; DAPT *n*=5/5; Fig. 2H-I). As expected, *cHairy2* was completely lost in floor plate and Hensen's node explants following DAPT treatment (controls *n*=21/22; *n*=20/22: treated *n*=25/25; *n*=16/18, respectively; supplementary material Fig. S1A-D). I-LNPs alone failed to express *cNetrin1*, Isl1 or Foxa2 (*n*=18/18; *n*=32/32, respectively; Fig. 2D,G,J). These data suggest that Notch is required for notochord-mediated induction of floor plate but not motor neurons.

DAPT did not affect *cBrachyury* (*cT*) or 3B9/cNot1 expression in co-cultures (*n*=12/13; controls *n*=14/14; Fig. 2M,N; data not shown) or node- or notochord-only explants (*n*=8/9 and *n*=12/12; controls *n*=8/8 and 16/16; supplementary material Fig. S1G-J). These data indicate that Notch acts specifically within the neuroectoderm to modulate the response of this tissue to Shh. Floor plate induction requires a higher dose of Shh than motor neuron induction and, in the absence of Notch, the neuroectoderm displays only the low-dose response to Shh.

The TUNEL assay did not reveal a significant difference in apoptotic index between DAPT and control explants (controls *n*=3, DAPT *n*=3; one-way ANOVA, d.f.=1; *F*=1.235; *P*=0.274; supplementary material Fig. S2B,B′). However, DAPT explants were visibly smaller, likely due to the significant reduction in the mitotic index (reduced number of phospho-histone H3-labelled cells; controls *n*=2, DAPT *n*=2; Kruskal–Wallis one-way ANOVA, d.f.=1; H=14.286; *P*≤0.001; supplementary material Fig. S2A,A′), as expected in the absence of Notch activity.

### Notch modifies sensitivity to Shh

The finding that Notch inhibition blocks floor plate induction could be due to lower Shh production by notochord or to a higher concentration of Shh required by the neuroectodermal cells. To distinguish between these possibilities, we repeated the assay but substituted notochord with varying concentrations of ShhN protein in the presence/absence of DAPT. Controls cultured in 4 nM ShhN expressed both Foxa2 (*n*=12/12) and Isl1 (*n*=13/13; Fig. 2P,T). DAPT abrogated Foxa2 expression (*n*=16/16) but Isl1 persisted (*n*=17/20; Fig. 2Q,U). These data recapitulate the results observed with the notochord assay. I-LNP explants cultured in 8 nM ShhN plus DAPT expressed both Foxa2 (*n*=11/15) and Isl1 (*n*=15/15; Fig. 2R,V). As an excess of ShhN rescued floor plate induction, these data imply Notch acts in neural cells to lower their threshold response to Shh and thereby specify the cell fate they acquire.

### *cHairy2* misexpression leads to dorsal expansion of P3 and early floor plate markers

**Fig. 3.**
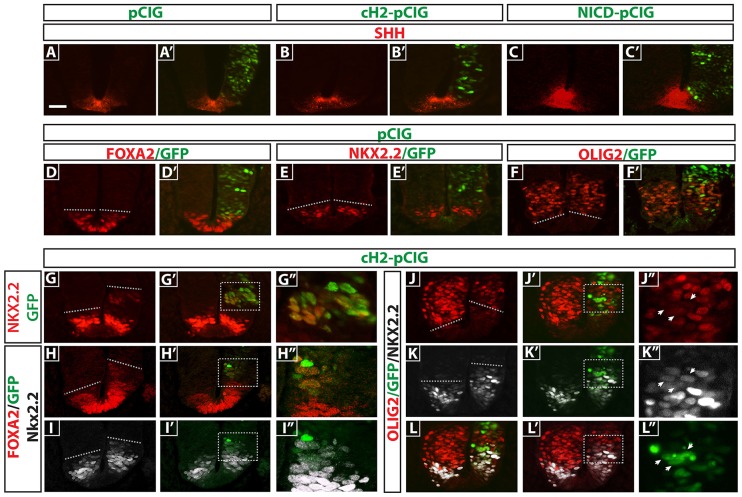
***cHairy2* misexpression dorsally expands P3 and early floor plate domains.** (A-L′) Sections of HH17 chick neural tube 24 h after electroporation with pCIG (A,A′,D,D′,E,E′,F,F′), pCIG-cHairy2 (B-C′,G-L′) or 48 h after pCIG-NICD electroporation (C,C′) analysed by immunohistochemistry for GFP (A-L′). Samples were also analysed for Shh (A-B′), Foxa2 (D,D′), Nkx2.2 (E,E′,G-G″) or Olig2 (F,F′) or double immunohistochemistry for Foxa2 and Nkx2.2 (H-I″) or Olig2 and Nkx2.2 (J-L″). (G″-L″) Magnified regions of interest are shown in G′-L′. Arrowheads in J″-L″ indicate three cells analysed for GFP, Nkx2.2 and Olig2. Scale bar: 30 µm.

To test whether Notch modifies the threshold concentration of Shh perceived via induction of Shh itself, we electroporated the caudal neural tube of HH stage 10 embryos with pCIG-NICD [pCAAGs vector encoding both a constitutively active form of Notch (Notch intracellular domain, NICD, normally only released following ligand-activated γ-secretase cleavage) and GFP, separated by an IRES] or the Notch target *cHairy2* [pCIG-cHairy2], and analysed Shh expression by immunohistochemistry. We observed by *in situ* hybridisation and qRT-PCR that NICD misexpression induces ectopic *cHairy2* expression in the neural tube (*n*=5, 75/93 sections; supplementary material Fig. S3; data not shown). However, neither NICD nor *cHairy2* electroporation altered the endogenous expression profile of Shh (*n*=5, *n*=3 embryos, respectively; Fig. 3A-C′). To ensure this was not due to cells having lost competence to acquire floor plate characteristics, we electroporated the open neural plate with pCIG-NICD at HH stage 6, cultured the embryos for 6 h and isolated GFP-positive I-LNP explants, then cultured these for 36 h; again, we saw no Shh induction (*n*=6; supplementary material Fig. S3). Thus, Notch signalling does not induce Shh expression.

We tested the hypothesis that *cHairy2* misexpression in more dorsal regions may induce the differentiation of more ventral characteristics by changing the sensitivity of those cells to the endogenous Shh morphogen gradient. *cHairy2* electroporation led to a dorsal expansion of the domains of Foxa2^+^ cells and Nkx2.2^+^ cells and a concomitant reduction of the domain of Olig2^+^ cells (*n*=2 for each marker pCIG; *n*=6 for each marker *cHairy2*; Fig. 3D-L″). Moreover, within the motor neuron progenitor domain, cells that downregulated Olig2 cell-autonomously upregulated Nkx2.2, indicating a change of fate from a motor neuron to a p3 progenitor (Fig. 3J-L″, *n*=6). Specification of ventral cell types is progressive: midline cells, which constitute the presumptive floor plate, initially express markers common to p3 progenitors, i.e. Foxa2 and Nkx2.2. Nkx2.2 becomes downregulated, and late FP markers, including Shh and Arx, are induced (Ribes et al., 2010). We used double immunohistochemistry to determine whether ectopic activation of Foxa2 by misexpression of cHairy2 is indicative of floor plate or p3 identity. We observed that the predominant response was upregulation of Nkx2.2 (*n*=2 embryos, 90/159 sections), with half those sections co-expressing Foxa2 (47 sections; Fig. 3H-I′). By contrast, definitive early floor plate fate (Foxa2^+^ only) was induced less robustly (Fig. 3H-I″).

In a complementary approach, we electroporated a dominant-negative form of *cHairy2* (lacking the WRPW domain; Broom et al., 2012) and observed downregulation of Nkx2.2 in the P3 domain where *cHairy2* is endogenously expressed at this stage (*n*=2; supplementary material Fig. S4). These findings imply that reducing Notch activity can increase the threshold concentration at which neural cells respond to Shh and thereby modify the extent of the expression domains of distinct dorsoventral markers induced by Shh. In particular, Notch activity, mediated by cHairy2, promotes acquisition of P3 identity and, to a lesser extent, early floor plate identity in response to Shh.

### Prolonged Notch activity/*cHairy2* expression in ventral midline cells prevents floor plate maturation and promotes P3 identity

**Fig. 4.**
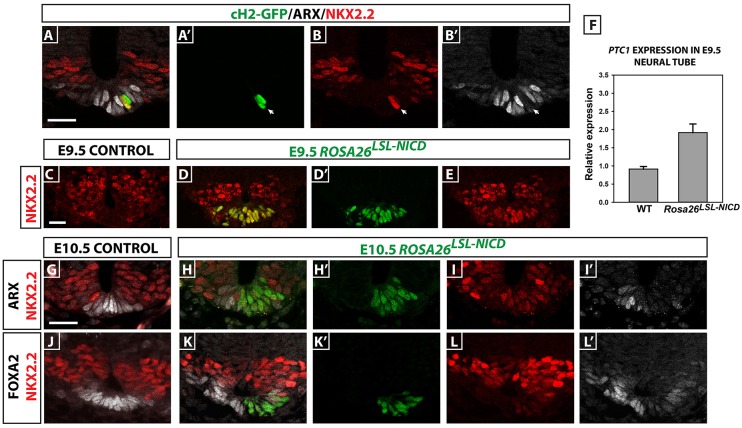
**Maintained Notch activity/*cHairy2* expression prevents floor plate maturation and promotes P3 identity.** (A-B′) Sections of HH17 neural tube 24 h after pCIG-cHairy2 electroporation into the ventral midline (cH2-GFP). Scale bar: 15 µm. (C-E) Sections of E9.5 or (G-L′) E10.5 *Foxa2^mcm^; Rosa26^LSL-NICD^* embryos. Scale bar: 30 µm. Sections analysed for GFP, Arx and Nkx2.2 (A-B′,G-I′) or GFP and Nkx2.2 (C-E) or GFP, Foxa2 and Nkx2.2 (J-L′). (F) qRT-PCR analysis of *Ptch1* mRNA levels in E9.5 *Foxa2^mcm^*; *Rosa^26LSL-NICD^* caudal neural tube compared with wild types. Bars represent mean values (plus s.e.m.) of relative expression levels from wild-type (0.913±0.078, *n=3*) and mutant embryos (1.918±0.239, *n=9*) normalised against β-actin.

*Ptch1* mRNA is only transiently expressed by floor plate as these cells attenuate their response to Shh to acquire full floor plate fate, in contrast to P3 progenitors that require sustained Shh signalling and maintain *Ptch1* expression (Ribes et al., 2010). *cHairy2* expression mirrors that of *Ptch1* in these domains. We tested the hypothesis that *cHairy2* too must be extinguished in ventral midline cells for them to acquire full floor plate fate. Embryos electroporated with *cHairy2* in the ventral midline at HH10 and harvested at HH17 displayed a cell-autonomous exclusion of the mature floor plate marker ARX in targeted cells (*n*=3 embryos, Fig. 4A-B′) with a concomitant upregulation of Nkx2.2 in some cases, indicating a fate change to P3 identity (Fig. 4A-B′). These data demonstrate that *cHairy2* can modulate the response of cells to Shh and imply that *cHairy2* is necessary for the acquisition of ventral cell fate in response to high Shh signal concentration but it also needs to be downregulated for floor plate cells to fully mature and differentiate. We next investigated whether loss of Notch activity is also necessary for full acquisition of floor plate fate. To achieve this, we used a conditional mouse line in which NICD is persistently expressed in the floor plate [tamoxifen-inducible mER;Cre;mER recombinase driver line under the control of the Foxa2 promoter (Foxa2^mcm^) crossed with a *Rosa^26LSL-NICD^* line (Murtaugh et al., 2003; Park et al., 2008)]. The *Rosa^26LSL-NICD^* strain permits conditional expression of NICD in cells expressing Cre recombinase. Strikingly, in these E9.5 and E10.5 embryos, we observed a cell-autonomous upregulation of Nkx2.2 in lineage labelled ventral midline cells (Fig. 4C-E,G-L′) concomitant with a downregulation of both Foxa2 and Arx (*n*=6 embryos; Fig. 4G-L′). These results phenocopy *cHairy2* electroporation in the chick floor plate. They suggest that elevated Notch activity is sufficient to maintain competence to respond to Shh in the ventral midline, supporting a role for Notch in the induction/maintenance of the p3 fate. Strikingly, qRT-PCR for *Ptch1* in caudal neural tissue isolated from E9.5 *Foxa2^mcm^; Rosa^26LSL-NICD^* embryos revealed *Ptch1* mRNA levels were doubled in the neural tube of these embryos (Fig. 4F; *n*=13) when compared with wild-type siblings, demonstrating that Notch activity augments the cellular response to Shh.

### Notch modifies the cell fate choice of neural progenitors in response to Shh *in vivo*

**Fig. 5.**
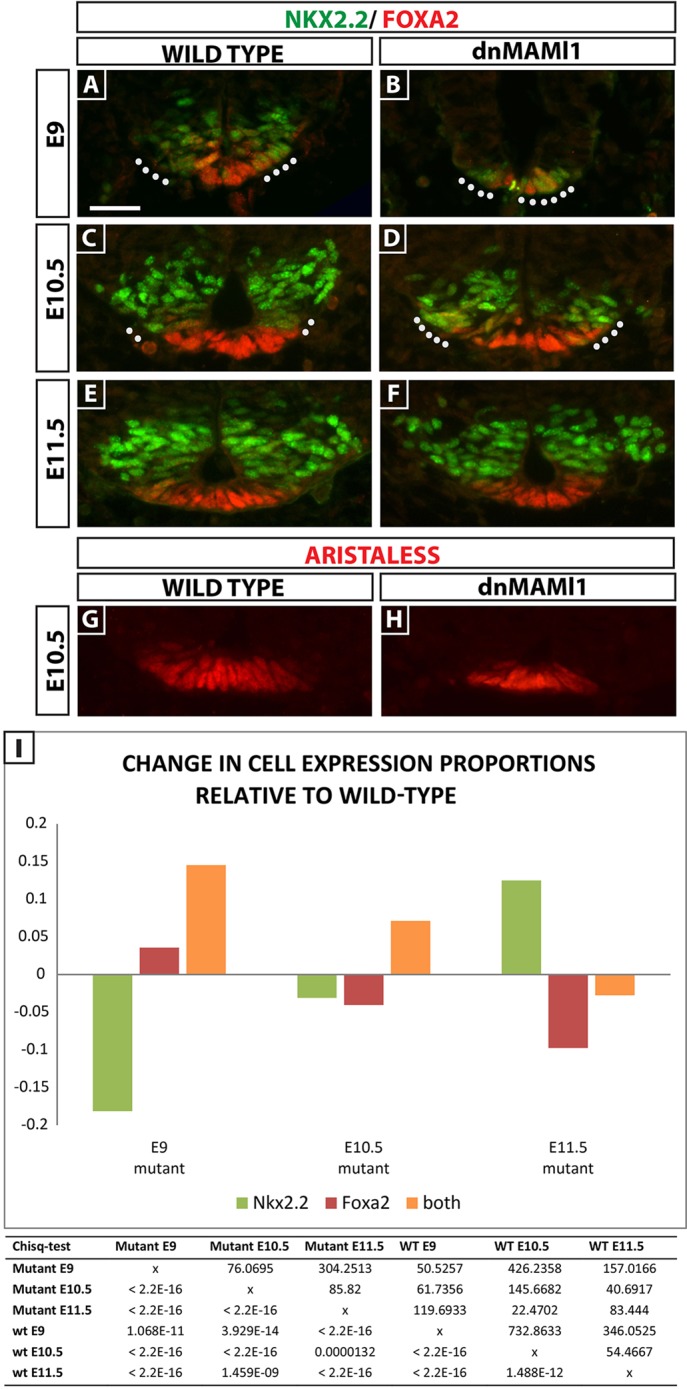
**Loss of Notch in the ventral midline in *Foxa2^T2AiCre^; Rosa26^LSL-dnMaml1^* mice modifies cell fate.** (A-H) Sections of spinal cord at lumbar level of E9 (A,B), E10.5 (C,D,G,H) or E11.5 (E,F) *Foxa2^T2AiCre^; Rosa26^LSL-dnMaml1^*mutants. Scale bar: 30 µm. (A-F) Sections analysed for Foxa2 and Nkx2.2. (G,H) Sections analysed for Arx. (I) Changes in proportions of cells positive for either Foxa2 (red, early floor plate), Nkx2.2 (green, P3 progenitor) or Foxa2^+^/Nkx2.2^+^ (orange, midline cells that may become floor plate or P3) within the ventral neural tube of E9, E10.5 or E11.5 *Foxa2^T2AiCre^; Rosa26^LSL-dnMaml1^* mutants. The number/location of Foxa2^+^/Nkx2.2^+^ cells (white dots in A-D) changes in the mutant at E9 and E10.5. The table presents the results of a statistical comparison between wild-type and mutant embryos of the three cell types using a Chi-square test. The distribution of cells within these three cell types was statistically different in the mutant compared with wild type at all three developmental stages. Chi squared values are in the top right of the table and *P*-values are at the bottom left of the table. All pairwise comparisons were highly statistically significant. Sections counted at E9: wild type, *n*=40; mutant, *n*=16; at E10.5, wild type, *n*=25; mutant, *n*=16; at E11.5, wild type, *n*=32; mutant, *n*= 25.

To determine whether Notch is necessary for neural tube patterning in response to Shh, we analysed the same progenitor markers in presenilin (*Psen*) *Psen1^−/−^; Psen2^−/−^* and *Rbpj^−/−^* embryos. Presenilins 1 and 2 are the key components of the γ-secretase enzyme complex that cleaves NICD. Thus, *Psen1^−/−^; Psen2^−/−^* embryos lack all Notch signalling, whereas *Rbpj^−/−^* embryos lack the obligate transcription factor required for Notch signalling. Both phenotypes are embryonic lethal at E9.5 (Oka et al., 1995; Donoviel et al., 1999). In both lines, the combined expression domains of Foxa2 and Nkx2.2 in the ventral neural tube were dramatically reduced at E9 (*n*=6 heterozygous controls and *n*=3 *Psen1^−/−^; Psen2^−/^*^−^; *n*=3 heterozygotes and *n*=3 *Rbpj^−/−^*; supplementary material Fig. S5; data not shown). In *Rbpj^−/−^* embryos, cell counts showed the domain of the caudal neural tube occupied by Foxa2^+^/Nkx2.2^+^ cells to be significantly lower than in controls (4% versus 7% in heterozygotes; *P*<0.001). To examine mutant embryos that survive beyond E9.5, we analysed mice in which the transcriptional activity of Notch is blocked only in ventral midline and P3 progenitors using Foxa2^T2AiCre^-induced expression of a dominant-negative Mastermind-like1 eGFP fusion protein from a targeted *Rosa26* locus (*Rosa26^dnMaml1^*; Fig. 5; High et al., 2008; Tu et al., 2005; Horn et al., 2012; Maillard et al., 2008). Dominant-negative MAMl1 eGFP fusion protein is a potent and specific inhibitor of all four mammalian Notch receptors *in vivo*. *Foxa2^T2AiCre^; Rosa26^dnMaml1^* embryos analysed at E9 (*n*=3) revealed that, within the combined Foxa2^+^, Nkx2.2^+^ or Foxa2^+^/Nkx2.2^+^ domain, most cells were Foxa2^+^/Nkx2.2^+^, indicative of an immature FP and/or P3 cell type (*n*=5; Fig. 5A,B), when compared with controls (Foxa2T2AiCre; Rosa26RYFP stage-matched embryos in which the Cre-recombined cells are normally Notch sensitive; data not shown). Quantification of these effects revealed a significantly higher proportion of Foxa2^+^/Nkx2.2^+^ cells and a significantly lower proportion of P3 (Nkx2.2^+^ only) cells (Fig. 5I; *χ*^2^=50.52, d.f.=1, *n*=2351 cells, *P*=1.068×10^−11^). The number of Foxa2^+^-only cells is relatively low in both controls and mutants at this developmental stage. E10.5 *Foxa2^T2AiCre^; Rosa26^dnMaml1^* embryos also showed a significantly different distribution of these three cell types when compared with controls, in particular maintaining a higher proportion of Foxa2^+^/Nkx2.2^+^ cells and a lower proportion of P3 (Nkx2.2^+^ only) and early floor plate cells (Foxa2^+^ only) (Fig. 5C,D; *χ*^2^=145.66, d.f.=1, *n*=2462 cells, *P*≤2.2×10^−16^). At E11.5, a significantly different distribution of cells in these three categories was maintained (Fig. 5I; *χ*^2^=83.44, d.f.=1, *n*=3151 cells, *P*≤2.2×10^−16^); while the proportion of double-positive cells was similar to controls (Fig. 5E,F,I), these embryos exhibited a significantly higher proportion of P3 and lower proportion of floor plate cells compared with controls (Fig. 5I). Analysis of Arx and Shh expression at E10.5 reveals that this small population of early floor plate cells go on to express late floor plate markers in the absence of Notch (Fig. 5G,H; data not shown). These data suggest that in the absence of Notch signaling in the ventral progenitors, the adoption of P3 or floor plate fates is delayed. Eventually, this resolves such that significantly fewer cells give the high dose response.

### Notch activity regulates localisation of Smo to the primary cilia in a Shh-independent manner

**Fig. 6.**
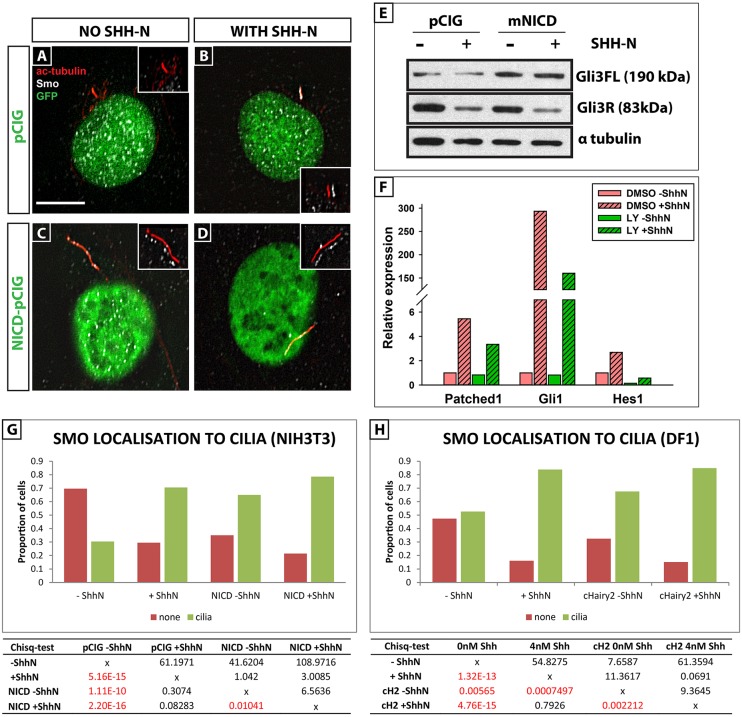
**Notch promotes cilia localisation of Smo in a Shh-independent manner.** (A-D) NIH3T3 cells transfected with pCIG-GFP (A,B) or NICD-pCIG-GFP (C,D) in the presence (B,D) or absence (A,C) of Shh-N. green, pCIG-GFP or NICD-pCIG-GFP; red, cilia labelled with α-acetylated tubulin; white, Smo antibody staining. Scale bar: 8 µm. (E) Western blot showing levels of full-length Gli3 (Gli3FL) and partially proteolysed repressor form (Gli3R) in cells transfected with pCIG-GFP or NICD-pCIG-GFP in presence or absence of ShhN. (F) qRT-PCR analysis of *Ptch1*, *Gli1* and *Hes1* mRNA levels in NIH3T3 cells in the presence of DMSO, DMSO+Shh-N or LY+Shh-N. Data shown are the mean from two biological replicates. (G,H) Quantification of NIH3 T3 (G) or DF-1 cells (H) depicting the proportions of cells that showed no Smo in cilia (red) versus cells that showed Smo localization in cilia (green) under different conditions. Data show proportion of cells with or without Smo localised to the primary cilium, and were compared using a Chi-square test. Cells transfected with NICD-pCIG-GFP had significantly longer cilia than pCIG-GFP transfected cells independent of ShhN (compare C,D with A,B). The table presents statistical analysis of effects on smo localisation to the cilia in 3T3 cells (table on the left hand) and DF-1 cells (table on the right hand). All pairwise comparisons made with 1 degree of freedom. Significant differences after Bonferroni correction are highlighted in red. Chi squared values are in the top right of the table and *P*-values are at the bottom left of the table. Significant results are in red.

Ciliary localisation of Smo is crucial for activation of the Shh pathway (Huangfu et al., 2003; reviewed by Sasai and Briscoe, 2012). To test whether regulation of Smo localisation might be a mechanism through which Notch activity modulates the efficacy of Shh signalling, we used the Shh-responsive NIH-3T3 primary fibroblast cell line. Double-labelling with antibodies to Smo and to acetylated tubulin (which marks stable microtubules found in cilia) revealed a highly significant accumulation of Smo in the cilia of NIH-3T3 cells upon addition of ShhN (Fig. 6B,G; *χ*^2^=61.19, d.f.=1, *n*=157 cells, *P*=5.16×10^−15^) whereas, in the absence of ShhN, cilia are largely devoid of Smo (Fig. 6A,G) (Rohatgi et al., 2007). Strikingly, activation of Notch, by transfecting NIH-3T3 cells with NICD-pCIG, dramatically and significantly augmented the number of cells showing Smo localisation in cilia, in a Shh-independent manner (Fig. 6C,G; *χ*^2^=41.62, d.f.*=*1, *n*=139, *P*=1.11×10^−10^). Addition of ShhN to the NICD-pCIG-transfected cells further enhances the effect (Fig. 6D,G; *χ*^2^=6.56, d.f.=1, *n*=130, *P*=0.01041). To preclude the possibility of a cell-type specific effect, we replicated the experiments in chicken DF-1 fibroblast cells by transfecting cells with *cHairy2-pCIG*, to closely mimic the electroporation analysis performed in chick embryos. We observed a very similar phenotype, whereby cHairy2-pCIG transfection dramatically increased the proportion of cells with SMO localisation in cilia, again in a Shh-independent manner (Fig. 6H; *χ*^2^=7.65, d.f.=1, *n*=116, *P*=0.00565). The further addition of ShhN augmented this effect (Fig. 6H; *χ*^2^=9.36, d.f.=1, *n*=73, *P*=0.002212). In the absence of Shh, Ptch1 inhibits Smo localisation in primary cilia. We measured intensity of Ptch1 staining in cilia of NIH-3T3 cells by double-labeling with antibodies to Ptch and Arl13b (a small GTPase that localises to cilia; see Caspary et al., 2007), and saw a dramatic reduction when we added ShhN, as expected (supplementary material Fig. S6). To address whether Notch modulates this effect, we treated NIH-3T3 cells with 150 nM LY411575, a γ-secretase inhibitor, which abolished *Hes1* mRNA expression, as expected (Fig. 6F; Ferjentsik et al., 2009). Remarkably, Notch inhibition led to a highly significant dampening of Ptch1 clearance from cilia in response to ShhN (*n*=2 replicates; 1205 cells). These data are reflected in the significant post-hoc pairwise comparisons using Tukey's HSD test [F(2,1205)=176.5, *P*≤0.001; supplementary material Fig. S6]. Taken together, these data demonstrate that Notch activity has a conserved, Shh-independent and significant effect upon localisation of key components of the Shh pathway within primary cilia, functioning at the level of the Ptch1/Smo interface to modulate ciliary localisation of these two signalling components.

### Notch signalling regulates the level of full-length Gli3 in NIH-3T3 cells

Full activation of Smo is a two-step process and ciliary transport is the initial requirement (Rohatgi et al., 2009, 2007; Sasai and Briscoe, 2012; Chen et al., 2011; Milenkovic et al., 2009). Within cilia, Smo can exist both in an inactive state and in the phosphorylated active form. The latter prevents proteolytic cleavage of the Gli transcription factors that then translocate to the nucleus to replace the cleaved repressor form and activate transcription of target genes (Stamataki et al., 2005; Briscoe and Thérond, 2013). Addition of 4 nM ShhN to NIH-3T3 fibroblasts leads to a sharp reduction in levels of Gli3R (*n*=3; Fig. 6E; Humke et al., 2010; Niewiadomski et al., 2014). We did not observe a concomitant increase in levels of full-length Gli3, probably due to the fact that this highly labile transcriptional activator undergoes rapid nuclear translocation, phosphorylation and destabilization (Humke et al., 2010). By contrast, exposure to NICD-pCIG or Hes1-pCIG alone led to an increase in the levels of full-length Gli3, in NIH-3T3 fibroblasts (*n*=3 replicates; Fig. 6E; data not shown). This is a striking result, given the 20% transfection efficiency (analysed by flow cytometry; data not shown). The addition of both ShhN and NICD-pCIG did not change levels of full-length Gli3 from that seen with NICD/Hes1 alone, although levels of Gli3R were dramatically reduced (*n*=3 replicates; Fig. 6E). These data reveal that concomitant with accumulating Smo protein in cilia, Notch signalling elevates levels of full-length Gli3. We next determined whether loss of Notch would affect Shh target gene transcription in NIH-3T3 fibroblasts. qRT-PCR analysis revealed 150 nM LY411575 dramatically reduced ShhN-mediated induction of *Ptc1* and *Gli1* (*n*=2 replicates; Fig. 6F). Notably, ShhN also induced *Hes1* expression in a Notch-dependent fashion, supporting the I-LNP data (Fig. 2B). Together, these data support the hypothesis that Notch signalling amplifies the cellular response to Shh.

### Notch signalling regulates cilia length both in the ventral neural tube and in NIH-3T3 fibroblasts

**Fig. 7.**
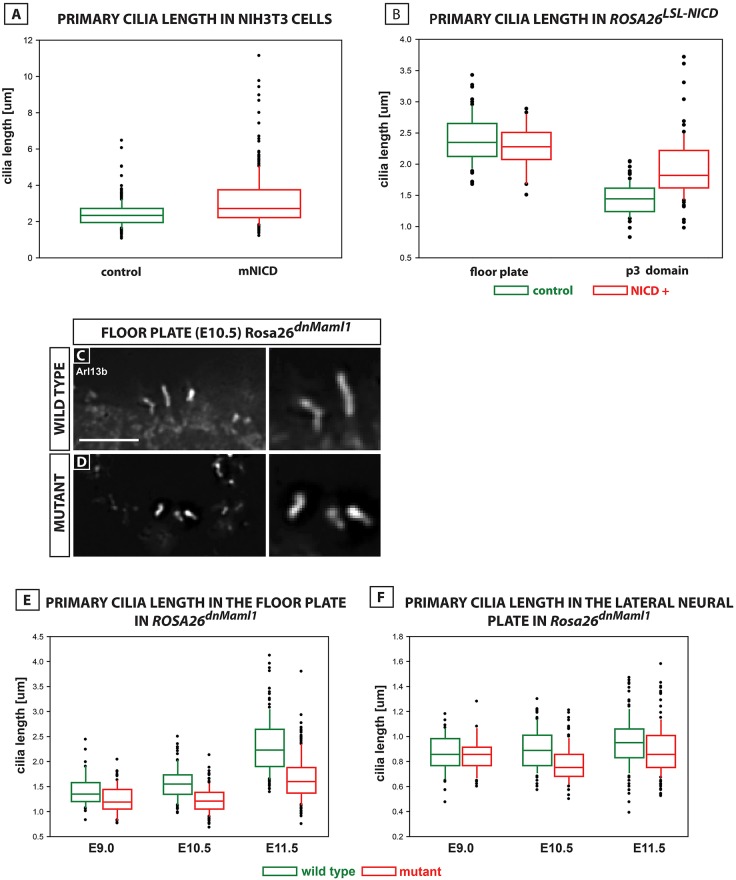
**Notch regulates cilia length.** (A,B) Quantitation of cilia length; box plots show median and range in NIH3t3 cells in presence/absence of NICD-pCIG-GFP (A), in floor plate and P3 domain of *Foxa2^mcm^; Rosa26^LSL-NICD^* mutants (NICD+) versus control littermates (B). (C,D) Cilia labelled with Arl13b antibody in the floor plate of *Foxa2^T2AiCre^; Rosa26^LSL-dnMaml1^* mutant (D) versus wild-type littermate (C). Scale bar: 5 µm. (E,F) Quantitation of cilia length in floor plate and lateral neural plate of *Foxa2^T2AiCre^; Rosa26^LSL-dnMaml1^* mutants (E9 *n*=3, E10.5 *n*=3 and E11.5 *n*=2) versus controls (E9 *n*=5, E10.5 *n*=2 and E11.5 *n*=2). Box plots show median and range; analysed by ANOVA. There is a highly significant three-way interaction between cell type (floor plate and P3), developmental stage (E9, 10.5, 11.5) and genotype (mutant/control) at the *P≤0.01 level [*F*(2,1354)=5.27, *P*=0.00525].

An additional striking and unexpected effect of NICD in NIH-3T3 cells is that cilia were significantly longer at the *P*≤0.001 level [*F*(2,1534)=128.557, *P*≤2.2×10^−16^; Fig. 6C,D compared with 6A,B; Fig. 7A]. *In vivo*, previous reports have shown the floor plate cilia become significantly longer than P3 cilia (Cruz et al., 2010; Fig. 7). To assess whether the effects of Notch upon ventral neural tube patterning may be associated with changes in cilia length, we measured cilia in the floor plate and lateral neural plate in both gain- and loss-of-function transgenic mouse models (*Foxa2^T2AiCre^; Rosa26^dnMaml1^* and in *Foxa2^mcm^; Rosa26^LSL-NICD^* mice). Using Arl13b antibodies, we observed that floor plate cilia in *Foxa2^T2AiCre^; Rosa26^dnMaml1^* embryos are significantly shorter than in control litter mates at E9, E10.5 and E11.5 at the *P*<0.01 level (E9 *n*=3 embryos, E10.5 *n*=3 embryos, E11.5 *n*=2 embryos; 1354 cells counted; Fig. 7C-F; floor plate: *P*=0.003682, *P*<2.2×10^−16^, *P*<2.2×10^−16^, respectively). These data are reflected in the significant post-hoc pairwise comparisons using the Tukey's HSD test in Table 1. By contrast, when we analyse cilia length in the floor plate and P3 domain in gain-of-function *Foxa2^mcm^; Rosa26^LSL-NICD^* mice at E10.5, we observed P3 cilia were significantly longer (0.4 µm more) than in control siblings (*n*=2 embryos; 249 cells; Fig. 7B; *P*≤2.2×10^−16^; Table 1). Floor plate cilia, however, were no different in length compared with controls (Fig. 7B; *P*=0.5624687; Table 1). These observations suggest that the mechanism by which Notch modulates the response of cells to Shh might be through regulating both cilia length and localisation of Smo within the cilia.

**Table 1.**
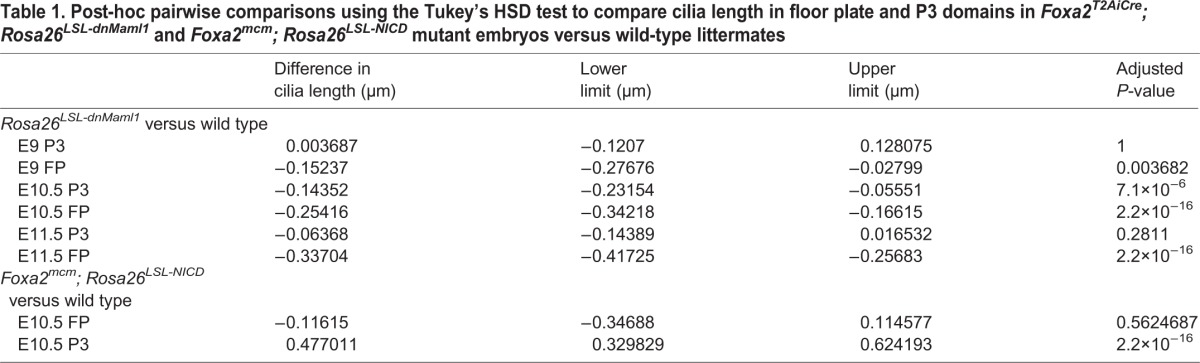
**Post-hoc pairwise comparisons using the Tukey's HSD test to compare cilia length in floor plate and P3 domains in *Foxa2^T2AiCre^; Rosa26^LSL-dnMaml1^* and *Foxa2^mcm^; Rosa26^LSL-NICD^* mutant embryos versus wild-type littermates**

## DISCUSSION

Primary cilia are important regulators of Shh signal transduction (Sasai and Briscoe, 2012). We provide *in vitro* and *in vivo* evidence supporting a novel role for the Notch pathway in modulating ciliary architecture and localisation of Smo to this cell appendage. We propose that this serves as a means to prime cells to respond to the Shh morphogen. *In vivo*, this synergistic interaction plays a key part in dorsoventral patterning of the developing neural tube in both chick and mouse embryos.

Intriguingly, NICD production occurs throughout the dorsoventral axis of the neural tube, yet the Notch target *cHairy2* expression is restricted to the floor plate/P3 domain (and the roof plate), in contrast to the broader expression of other Hes homologues (Fior and Henrique, 2005). We propose this is probably due to cooperative activation of *cHairy2* by Shh and Notch in the ventral midline. One possible explanation for how this synergy might operate is that Shh might induce expression of Notch itself or a Notch ligand. Indeed, NICD electroporation in more dorsal regions drives ectopic *cHairy2* expression in the neural tube indicative of the fact that endogenous levels of Notch signalling may be higher in the floor plate. Serrate 1 is a potential candidate in this respect, as it is expressed in the ventral midline of the chick and mouse neural tube at early developmental stages (J.K.D., unpublished; le Roux et al., 2003).

Paradoxically, we have shown previously that DAPT treatment of whole embryos completely blocked *cHairy2*, but did not alter Foxa2 expression (Gray and Dale, 2010), whereas we show here that DAPT treatment of I-LNPs blocks Shh induction of both *cHairy2* and Foxa2. However, in the whole embryo assay, we demonstrated a larger notochord forms from node progenitors in the absence of Notch. This increase in the number of Shh-producing cells and potentially the levels of Shh was proposed to mediate the induction of Foxa2 expression/floor plate characteristics in the absence of Notch. In support of that idea, we show in this study that rescue of Foxa2 in the absence of Notch can be achieved by simply increasing concentration of Shh to which the I-LNP is exposed.

We use a wide variety of gain- and loss-of-function approaches in both chicken and mouse to show that interactions between Notch and Shh are crucial for dorsal-ventral cell fate specification in the neural tube. Inhibiting Notch signalling impedes floor plate induction whereas ectopic Notch activation in more dorsal regions ventralises those cells. After floor plate induction, Notch signalling is normally downregulated in the ventral midline, and forced maintenance prevents floor plate maturation, resulting in the formation of more P3 progenitors in both chick and mouse at the expense of floor plate. This novel role for Notch is very different from the well-established role for Notch in maintaining neural progenitors and preventing their differentiation. We provide multiple lines of evidence that these effects are due to Notch modulating progenitor cell interpretation of the Shh spatiotemporal gradient rather than directly regulating cell fate or differentiation: first the *in vitro* I-LNP assay shows Notch inhibition changes sensitivity but not competence of cells to respond to Shh; second the *Foxa2^T2AiCre^; Rosa26^dnMaml1^* embryos show a temporal delay in competence to respond to Shh; third Notch modulation of Smo/Ptch1 trafficking to cilia, levels of Gli3 and Shh target gene expression *in vivo* and *in vitro* directly demonstrates that Notch affects progenitor cell interpretation/response to Shh. Although the dorsoventral patterning output of this Notch/Shh synergistic interaction has not been previously reported, an intriguing parallel with our findings is that, in zebrafish lateral floor plate progenitors, loss of Notch leads to loss of Hh response and initiation of Kolmer–Agduhr interneuron differentiation (Huang et al., 2012), although the mechanism governing this synergistic interaction remains unknown.

We show part of this mechanism relies on the subcellular localisation of the key Shh signalling component Smo; NICD misexpression *in vitro* dramatically augments the initial step of ciliary accumulation of Smo, in a Shh-independent manner. This effect is phenocopied by cHairy2 and thus is likely to be a transcriptional response. The identification of the NICD/cHairy2 target gene involved in trafficking Smo to the cilia will require further investigation.

In addition, NICD misexpression led to elevated levels of full-length Gli3 *in vitro*. This might be due to Notch promoting inhibition of the cleavage of full-length Gli to the repressor form directly or indirectly through increasing accumulation of Smo in cilia, and/or this may be a transcriptional response, given that Gli2 and Gli3 have previously been reported to be direct targets of N1ICD/Rbpj (Li et al., 2012). As expected, exposure to ShhN dramatically lowers levels of Gli3R, both in the presence/absence of NICD, which is not phenocopied by exposure to NICD alone. These data suggest NICD-mediated ciliary accumulation of Smo and elevated levels of full-length Gli3 are not sufficient to stimulate full activation of the Shh pathway. Rather, we propose that these events prime cells to respond efficiently and in a timely fashion when they become exposed to Shh.

An additional unexpected and Shh-independent effect of NICD activation within NIH-3T3 fibroblasts was the formation of significantly longer primary cilia. This effect on ciliary length is also evident *in vivo* in transgenic lines that serve to activate or inhibit Notch activity specifically in the ventral midline. This effect is likely to be transcriptionally regulated as it is observed in *Foxa2^T2AiCre^; Rosa26^dnMaml1^* embryos. A recent report demonstrated that supernumerary cilia, resulting in more ciliary signaling surfaces, reduced Shh pathway transcriptional activation (Mahjoub and Stearns, 2012). By contrast, within the neural tube of *Arl13b* mutants that form cilia half the length of normal cilia, there is a failure to induce dorsoventral markers that are characteristic of the highest levels of Shh signalling, although expression of genes that require lower levels of Shh signalling continues (Caspary et al., 2007). Thus, Shh signalling is sensitive to cilia length, number and architecture. It has previously been shown that cilia length changes within the ventral midline of the developing neural tube during normal development (Cruz et al., 2010). Thus, floor plate cilia become almost double the length of those in the adjacent lateral neural tube and this has been associated with Shh-dependent onset of Foxj1 expression within the floor plate (Cruz et al., 2010). Indeed, Foxj1 misexpression is sufficient (but not necessary) for longer cilia in the neural tube. In a variety of developmental contexts, Foxj1 has been associated with production of motile cilia that are considerably longer than primary cilia (Cruz et al., 2010; Blatt et al., 1999; Chen et al., 1998; Stubbs et al., 2008; Tichelaar et al., 1999; Yu et al., 2008). A previous report linking Notch signalling to ciliary architecture came from zebrafish (Lopes et al., 2010). Kupffer's vesicle is enriched with long motile cilia, the function of which is key for onset of left-right asymmetry. Lopes et al. showed Notch signalling directly regulates ciliary length, and inefficiencies in Notch signal transduction result in shorter cilia and aberrations in left-right asymmetry. One target of Notch in this regard is Foxj1 (Lopes et al., 2010). Thus, it is possible that Foxj1 mediates the NICD-dependent changes to ciliary architecture in the ventral neural tube. Intriguingly, Cruz et al. suggest Foxj1 attenuates the response to Shh and that this is cilia dependent. It is noteworthy, however, that the subcellular localisation of Smo is not affected by Foxj1 misexpression*,* indicating that NICD-mediated changes in cilia length and Smo localisation might be differentially regulated (Cruz et al., 2010). Indeed, we did not observe changes in cilia length following cHairy2 or Hes1 misexpression, indicating that this effect is mediated by a different set of Notch target effectors. Thus, our working model proposes that NICD activity leads to both increased ciliary length and ciliary localisation of Smo, by two independent mechanisms, which together prime the cell for an accentuated response to the Shh ligand.

The delay in acquisition of ventral fates seen in neural progenitors expressing dnMAMl1-eGFP indicates a potential role for Notch in regulating the temporal response to Shh. It is well established that the spatiotemporal pattern of ventral marker induction in the neural tube reflects changes in both concentration and duration of Shh signalling over time. Our data invoke a model in which the role of Shh-dependent Notch activity is to prime cells to respond efficiently and appropriately to Shh by increasing length of cilia and accumulation of SMO within these structures, thereby facilitating rapid Shh-triggered activation of SMO once ligand is received.

In conclusion, our findings, using both *in vitro* and *in vivo* models, reveal a conserved and novel mechanism to refine and modulate the response repertoire and cell sensitivity to Shh during tissue development. This role for Notch may also affect a broad range of other pathways that are reliant on ciliary localisation of signalling components in a wide variety of developmental and disease contexts.

## MATERIALS AND METHODS

### Chick embryo

White Leghorn *Gallus gallus* eggs (Henry Stewart, Lincolnshire, and Winter Farm, Royston, UK) or GFP-expressing embryos [Roslin Institute, Midlothian (McGrew et al., 2004)] were incubated at 38.5°C in a humidified incubator to yield embryos between Hamburger–Hamilton (HH) stages 5 and 17, according to Hamburger and Hamilton, 1992.

### Mouse embryos

Wild-type CD1 mouse (*Mus musculus*) embryos were obtained at E8.5-E11.5, fixed for immunohistochemistry or *in situ* hybridisation. Genotyping of *Rosa26^dnMaml1^* (Horn et al., 2012), *Foxa2*^*mcm*^ (Park et al., 2008), *Rosa26^LSL-NICD^* (Murtaugh et al., 2003), *Psen1^−/−^; Psen2^−/−^* (Donoviel et al., 1999), *Rbpj^−/−^* (Oka et al., 1995) and *Rosa26^LSL-YFP^* (Srinivas et al., 2001) was carried out using PCR. *Rosa26^dnMaml1^* and *Rbpj^−/−^* mice were kept as heterozygotes. *Foxa2^mcm^; Rosa26^LSL-NICD^* embryos were obtained by crossing *Foxa2*^mcm^ male with *Rosa26^LSL-NICD^* female. Pregnant females were administered 8 mg of tamoxifen (Sigma, T5648) by oral gavage at E7.5 and E8.5 dpc with embryos collected at E10.5 or at E6.5 and E7.5 dpc with embryos collected at E9.5.

### Cell culture

NIH-3T3 mouse and DF1 chick fibroblasts were cultured in a humidified incubator and maintained at 37°C in an atmosphere of 5% CO_2_, in Dulbecco's modified Eagle's medium (DMEM; Life Technologies) supplemented with 10% heat-inactivated Newborn Calf Serum (NBCS; Life Technologies) or foetal bovine serum (FBS, Sigma), respectively. Cells were transfected using Lipofectamine LTX with Plus Reagent Kit (Invitrogen) either with control empty plasmid (PCIG; Megason and McMahon, 2002), PCIG-NICD or pCIG-cHairy2 (Dale et al., 2003) at final concentration of 1 μg/ml. For ciliated cell enrichment, transfected cells were maintained 24 h in DMEM supplemented with 0.5% NBCS or 0.5% FBS for NIH3T3 and DF1 (which enriches for cells in G1, when cilia formation predominantly occurs), respectively (Basten and Giles, 2013), before treating them with 4 nM recombinant ShhN protein (University of Dundee, UK) for 12 h.

### Explant culture

Explants isolated from HH stage 6 or 7 chick embryos were cultured in collagen (Placzek and Dale, 1999) in 100 μM γ-secretase inhibitor IX (DAPT) (Calbiochem) dissolved in dimethylsulphoxide (DMSO) (Sigma) or DMSO alone for 36 h unless stated otherwise. Notochord and intermediate lateral neural plate (I-LNP) co-culture explants employed notochord from a GFP-transgenic embryo to distinguish neural versus mesodermal expression of Foxa2 and Isl1. Where stated, I-LNP explants were cultured in 1 nM or 4 nM recombinant Shh protein.

### Electroporation

pCIG-NICD, pCIG-*cHairy2*, pCIG-dominant-negative *cHairy2* (Broom et al., 2012) or pCIG vectors were introduced to the caudal neural plate of HH10-12 embryos using standard *in ovo* electroporation (Dale et al., 2003; Briscoe et al., 2000). Embryos were cultured overnight before fixation.

### *In situ* hybridisation and immunohistochemistry

Standard *in situ* hybridisation methods were used (Henrique et al., 1995). Embryos were fixed in 4% PFA in PBS at pH 7.2, on ice for 2 h. Cells growing on coverslips were fixed in 4% PFA in PBS for 15 min. For patched 1 antibody, cells were fixed with 2% PFA for 5 min followed by 5 min of ice-cold methanol at −20°C. Antibody protocols have been described for Foxa2 (1:10; DSHB), 3B9, Nkx2.2 (1:10; DSHB), Isl1 (1:10; DSHB), Shh (1:10; DSHB), Olig2 (1:16,000; a generous gift from B. Novitch, UCLA, USA), aristaless (Arx, 1:1000; Jamel Chelly, IGBMC, Paris, France), Arl13b (1:3000; N295B/66, UC Davis/NIH NeuroMa, USA), Smo (1:3000; Abcam, ab38686), Ptch1 (1:750; University of Dundee, UK), acetylated tubulin (1:1000; Sigma, T7451), mNICD (1:200; Cell Signaling, D3B8), cNICD (1:2000; University of Dundee), anti-GFP (1:1000; Life Technologies, A6455) (Ericson et al., 1996, 1997, 1992; Gibb et al., 2009; Yamada et al., 1991; Caspary et al., 2007; Cruz et al., 2010; Bone et al., 2014; Huppert et al., 2005).

### Phospho-histone-H3 immunohistochemistry on explants

Fixed tissue was proteinase K (Roche) treated and fixed (4% formaldehyde in PBS, 2 mM EGTA, 0.1% glutaraldehyde (Sigma). Anti-phospho-histone-H3 antibody (Upstate) was added at a concentration of 10 μg/ml.

### Marker gene analysis in mutant embryos

Cell counts for each marker were performed on sections and expressed as a proportion of the domain covered by the two markers. Heterozygotes provided a characteristic template of neuronal patterning from which comparisons could be made with mutant littermates.

### Western blotting

Western blot analysis has been described previously (Bone et al., 2014). Ten micrograms of sample was loaded. Gli3 rabbit antibodies (a generous gift from Susan Mackem, Center for Cancer Research, Frederick, MD, USA) and mouse anti-tubulin (Abcam) were diluted 1:1000 and 1:5000.

### qRT-PCR

Total RNA was extracted from the caudal region (below forelimb) of E9.5 *Foxa2^mcm^; Rosa26^LSL-NICD^* using a Qiagen Micro Plus kit. cDNA was synthesised using SuperScript III reverse transcriptase (Life Technologies). qRT-PC was performed with Power SYBR Green Master Mix (Life Technologies) and reactions measured in a C1000 Thermal Cycler (Bio-Rad) under the following conditions: 95°C for 5 min, 40 cycles 95°C for 15 s and 60°C for 1 min. *Ptch1*, *Gli1* (Han et al., 2009), *Hes1* (Li et al., 2012) and *cHairy2* primers (F: 5′-CCGTACCCTGCAAGCCAGGTG-3′, R: 5′-GCCCATCA-GAGGCAAGCAGCA-3′) were described previously and normalised against β-actin (Ferjentsik et al., 2009; Ribes et al., 2010) using the Pfaffl equation (Pfaffl, 2001).

### Image acquisition/analysis

Fluorescent signal was acquired using a compound microscope (Leica DM5000 B), an Olympus IX70 deconvolution microscope or the Zeiss LSM-710 confocal microscope. Image analysis was carried out in deconvolved pseudo-coloured images using Volocity software or the open access software Fiji.

### Statistical analysis

Data analysis was conducted using the open source statistical software R (R Core Team, 2012). Differences in cilia length and PTC cilia intensity were tested using ANOVA on log transformed response data, with post-hoc pairwise comparisons conducted using Tukey's honest significant differences test. Differences in Smo protein localisation in the cilia in addition to changes in the counts of categories of cells expressing different markers across the anterior-posterior axis of the *Rosa26^dnMaml1^* or control mouse embryos were evaluated using a chi-square test. Plots were generated using R studio, sigma plot and Microsoft Office Excel. Phospho-histone-H3-labelled chromatin in control and treated explants was analysed by ANOVA.

## Supplementary Material

Supplementary Material
