# Supplementary material for: A conserved role for Notch signaling in priming the cellular response to Shh through ciliary localisation of the key Shh transducer Smo
Source: Development. 2015 Jul 1;142(13):2291–303. doi: 10.1242/dev.125237 (PMC4510595; doi:10.1242/dev.125237)
Supplement: Supplementary Material [file supp_142_13_2291__index.html]

Supplementary Material 

# A conserved role for Notch in priming the cellular response to Shh through ciliary localisation of the key Shh transducer, Smoothened

## DEV125237 Supplementary Material

- Supplementary Material
