# Supplementary material for: A conserved role for Notch signaling in priming the cellular response to Shh through ciliary localisation of the key Shh transducer Smo
Source: Development. 2015 Jul 1;142(13):2291–303. doi: 10.1242/dev.125237 (PMC4510595; doi:10.1242/dev.125237)
Supplement: Supplementary Material [file supp_dev.125237_DEV125237supp.pdf]

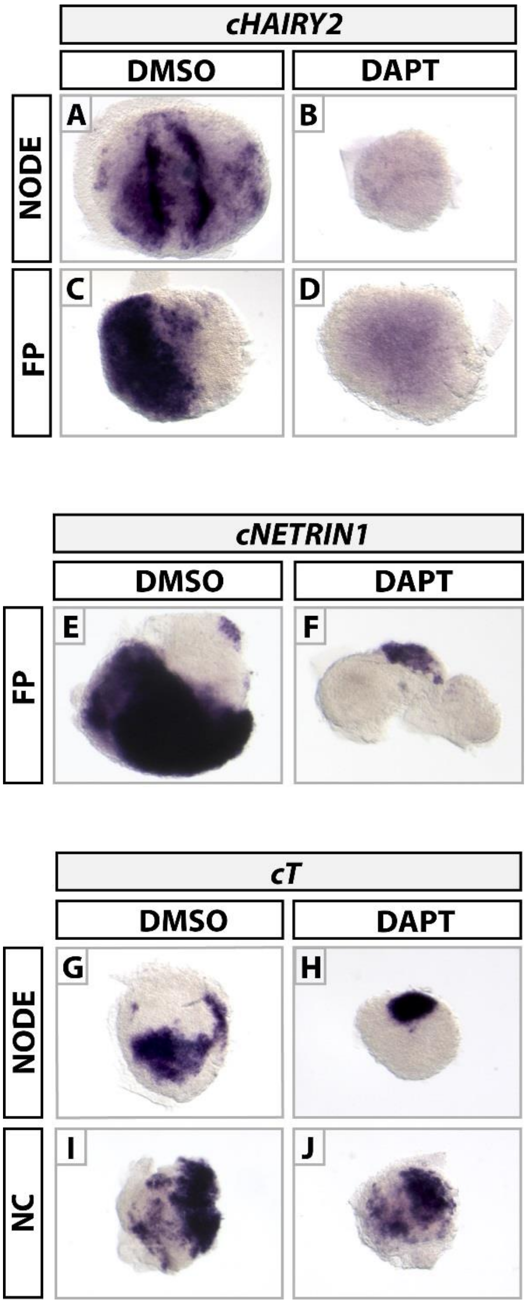

Supplementary Figure 1.

HH6/7 explants cultured in DMSO (A,C,E,G,I) or DAPT (B,D,F,H,J) analysed for *cHairy2* (A-D), *cNetrin1* (E-F), *cT* (G-J). *cHairy2* in Hensen's node (node) and floor plate (FP) (A,C) is down-regulated by DAPT (B,D). (E) FP cultured *in vitro* retains *cNetrin 1* expression, *n*=10/11. However, this is lost in the presence of DAPT (F). (I,J) Notochord (NC) expression of *cT* is unaffected by DAPT. *cT*; *cBrachyury*. Same magnification used for all images.

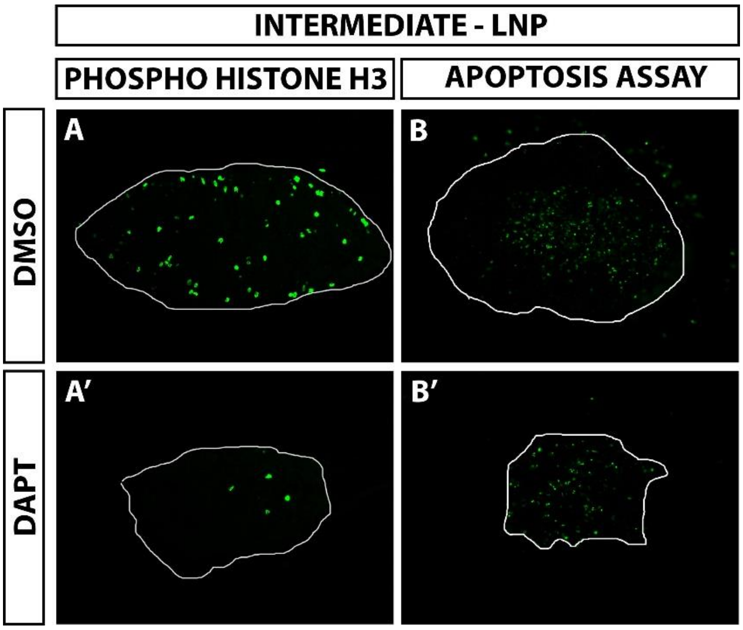

Supplementary Figure 2.

Sections from isolated neuroectoderm from HH6/7 embryos cultured in DMSO (A-B) or DAPT (A'-B'). Samples analysed with phospho histone-H3 antibodies (A, A') or the *In Situ* Cell Death Detection Kit (B, B').

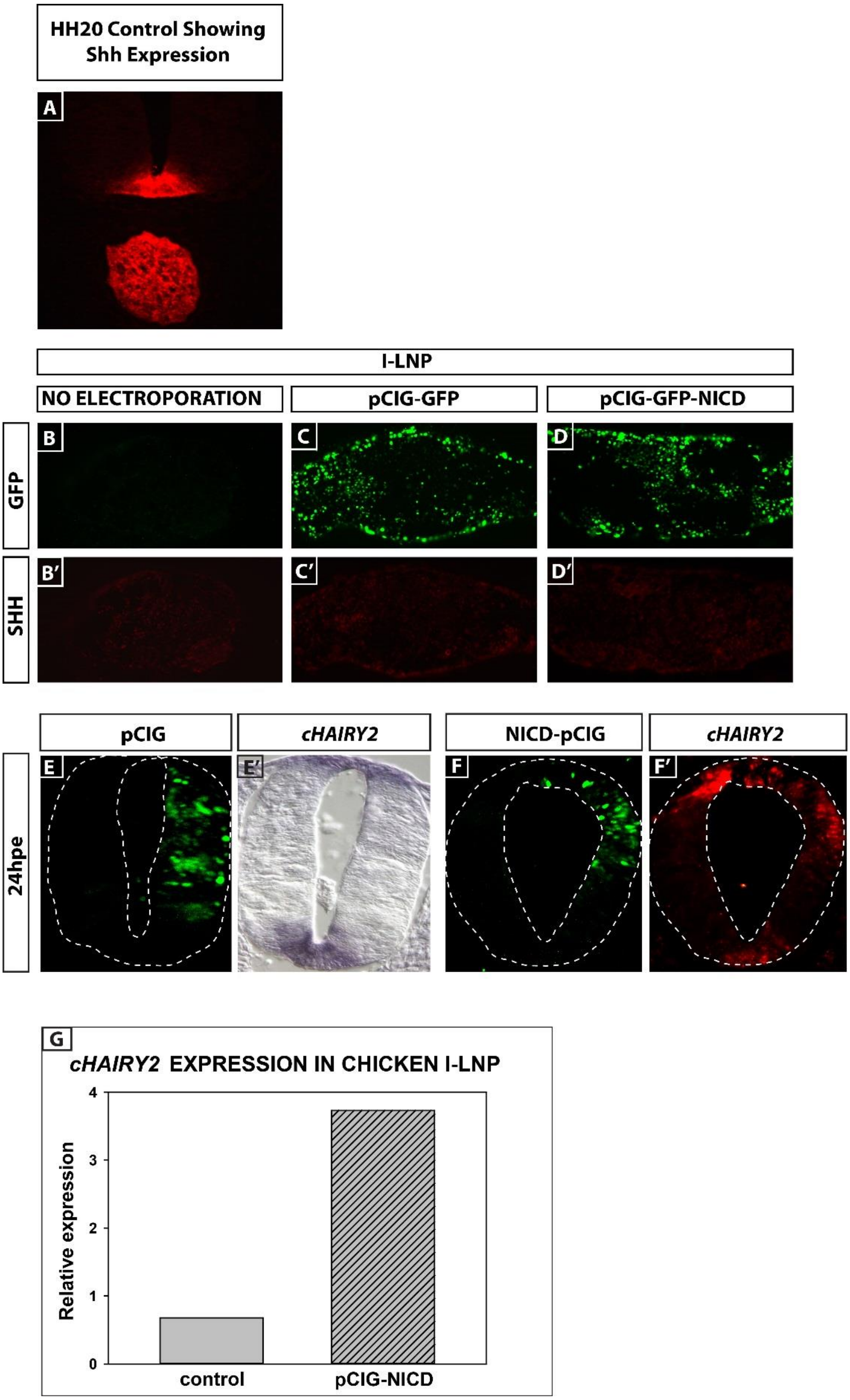

Supplementary Figure 3.

A) Shh expression in a transverse section of a control HH stage 20 chick embryo. B, B') A section of an isolated intermediate lateral neural plate (I-LNP) explant cultured alone, showing Shh is not

normally expressed by this tissue. C) A I-LNP section following electroporation with pCIG-GFP empty vector showing GFP-expressing cells throughout the explant. C') The same section as C, showing no Shh expression. D) A I-LNP section following electroporation with NICD-pCIG-GFP, showing GFP-expressing cells throughout the explant. D') The same section as D, showing that following NICD electroporation there is no induction of Shh. Sections were analysed by double immunohistochemistry for GFP and Shh expression. (E-F') Transverse sections of HH 17 chick neural tube 24 hours after electroporation with pCIG (E,E') or pCIG-NICD (F,F') analysed by regular or fluorescent in situ hybridisation for *cHairy2* expression. (G) qRT-PCR analysis of *cHairy2* mRNA levels, normalised to B-actin, in I-LNP explants electroporated with either control empty vector (pCIG) or pCIG-NICD.

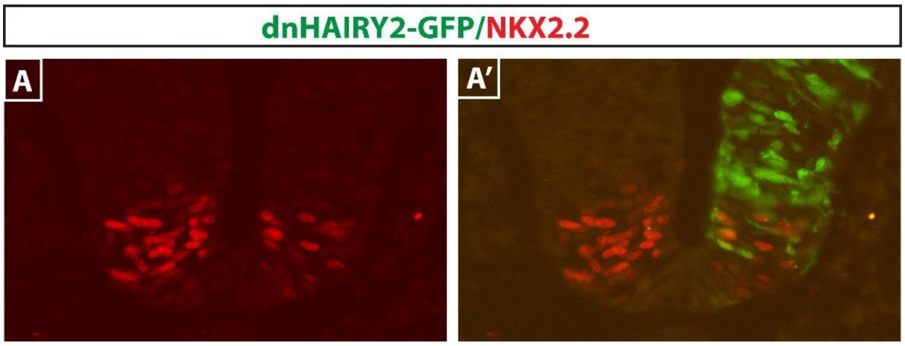

Supplementary Figure 4. (A-A') Transverse sections of HH 17 chick neural tube 24h after electroporation with pCIG-dominant-negative cHairy2 into the ventral midline (dnHairy2-GFP) (scale bar= 15µm). Sections were analysed for GFP and Nkx2.2 expression.

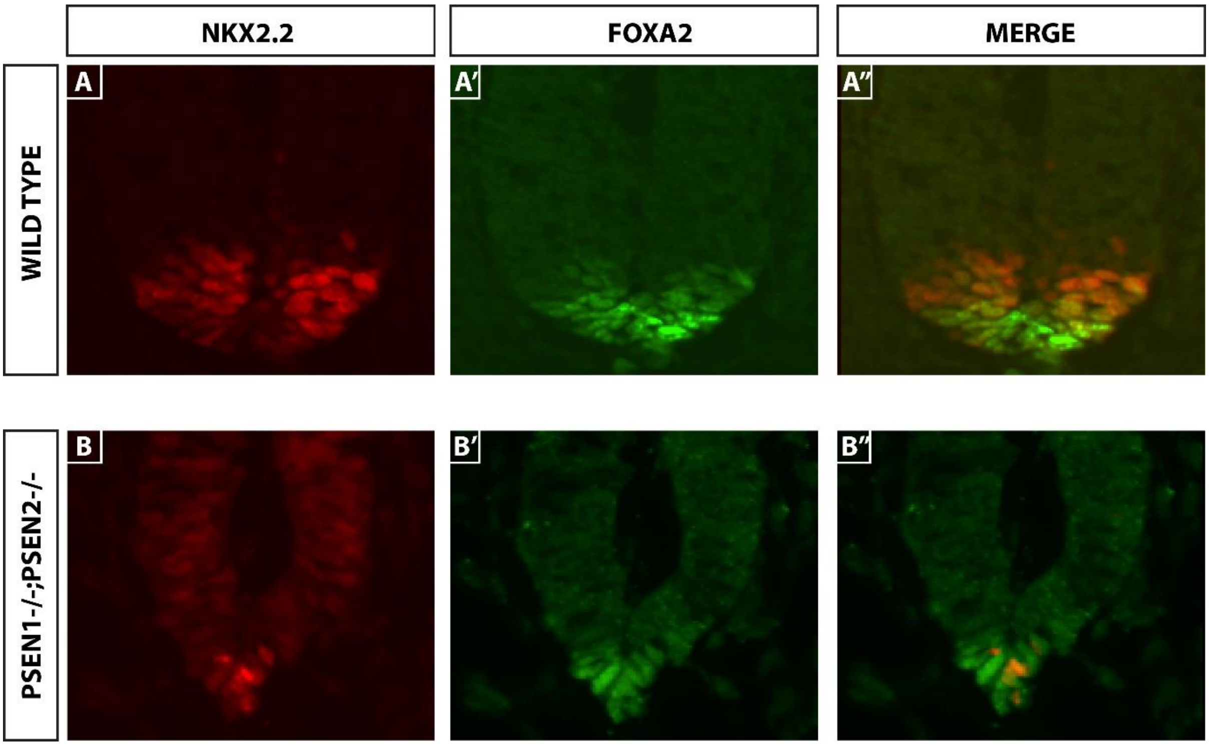

Supplementary Figure 5. (A-B') Transverse sections of neural tube of E9 *Psen1*$^{-/-}$; *Psen2*$^{-/-}$ mouse embryo (A-A'') and wild type litter mate (B-B''). Sections were analysed for Nkx2.2 and Foxa2 expression. The right-hand panels show the merged image.

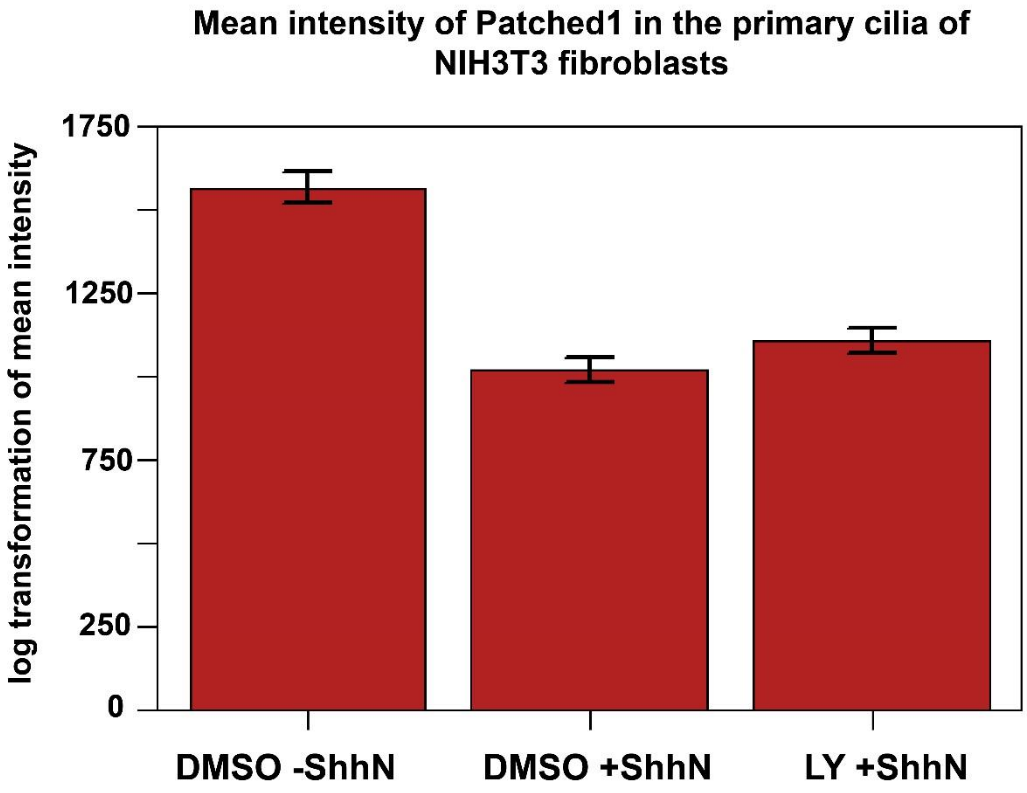

	df	Sum of squares	Mean square	F value	Significance
Between groups	2	45.43	22.716	176.5	$<2 \times 10^{-16}$
Within groups	1205	155.11	0.129		

	Difference	Lower bound	Upper bound	P value adjusted
DMSO +ShhN vs DMSO –ShhN	-0.45535195	-0.51532222	-0.3953817	<0.0005
LY +ShhN vs DMSO –ShhN	-0.36351561	-0.42303257	-0.3039987	<0.0005
LY +ShhN vs DMSO +ShhN	0.09183634	0.03320675	0.1504659	0.0007238

	Mean	95% ci
DMSO –ShhN	1566	+/- 45.3
DMSO +ShhN	1017	+/- 36.2
LY +ShhN	1109	+/- 37.8

Supplementary Figure 6. Bar chart plotting log transformation of PTC fluorescence intensity in cilia of NIH-3T3 cells cultured in DMSO, DMSO + 4 nM ShhN, or 150 nM LY + 4 nM ShhN. The region of interest was identified by expression of Arl13b, a specific marker of primary cilia. The min, max and mean intensity values for Patched1 signal were measured in Volocity software (PerkinElmer). For statistical analysis, the mean values were log transformed and compared using ANOVA (see below for table) and Tukey's Honest significant difference test (table can be seen below ANOVA table). There was a very highly significant effect for treatment, $F(2, 1205)=176.5$, $P<0.001$.
